# From commitment to capacity: strengthening Europe’s public health response to cardiovascular disease and diabetes through life-course prevention

**DOI:** 10.3389/phrs.2026.1609639

**Published:** 2026-06-24

**Authors:** Sarah Cuschieri

**Affiliations:** 1 University of Malta Faculty of Medicine and Surgery, Msida, Malta; 2 Department of Epidemiology and Biostatistic, Western University, London, ON, Canada

**Keywords:** Europe, life course epidemiology, non communicable diseases, policies, prevention

## Abstract

**Background:**

Cardiovascular disease and diabetes remain the leading causes of premature mortality in Europe, despite longstanding political commitments and the availability of cost-effective preventive interventions. As the global public health community approaches the 40th anniversary of the Ottawa Charter for Health Promotion, persistent gaps between prevention principles and implementation require renewed policy focus.

**Analysis:**

This policy brief synthesises evidence from international reports and European initiatives to examine why prevention efforts for cardiovascular disease and diabetes have not achieved sufficient scale or impact. It highlights the role of obesity, diabetes, and early-life risk exposure in shaping cardiovascular risk, alongside fragmented surveillance systems and continued reliance on reactive, treatment-centred care. While current policy frameworks demonstrate ambition, implementation and accountability remain inconsistent.

**Policy Options:**

Priority actions include embedding life-course prevention across cardiovascular and diabetes strategies, strengthening primary and community-based prevention, investing in equity-oriented surveillance, and scaling collaborative initiatives such as JACARDI and JA PreventNCD.

**Conclusion:**

Achieving equitable prevention of cardiovascular disease and diabetes requires a shift from reactive care to sustained life-course prevention.

## Background

Cardiovascular disease (CVD) and diabetes represent a substantial and persistent public health challenge in Europe. Together, they account for a large share of premature mortality, morbidity, and health-system expenditure, with disproportionate impacts on socioeconomically disadvantaged populations. Noncommunicable diseases (NCDs) are responsible for more than 90% of all deaths in the World Health Organization (WHO) European Region, with CVD as the leading cause and diabetes a major contributor to cardiovascular risk and long-term complications [1–3].

Despite repeated political commitments, the WHO Noncommunicable Diseases Progress Monitor 2025 demonstrates that implementation of recommended NCD prevention policies across Europe remains uneven, particularly for fiscal, regulatory, and prevention-oriented interventions [1]. Similarly, the European Health Report 2024 highlights persistent inequalities in premature mortality and insufficient progress towards prevention targets despite longstanding strategic commitments [2]. In fact, approximately 1.8 million NCD-related deaths occur annually before the age of 75 in Europe, and an estimated 60% of these deaths are preventable through effective action on modifiable risk factors [1, 2]. This is estimated to cost more than US$500 billion annually in lost productivity, with preventable deaths accounting for most of this burden [1, 2]. The COVID-19 pandemic further disrupted prevention, early detection, and chronic disease management, compounding existing challenges [4]. Therefore, continued underinvestment in prevention represents deferred expenditure transferred to future health systems, rather than cost containment. At the same time, the pandemic demonstrated that European health systems are capable of rapidly mobilising financial resources, regulatory flexibility, technological innovation, and coordinated multidisciplinary responses when faced with an acute health threat [3]. This contrast highlights an important policy challenge: while prevention operates over longer time horizons than infectious disease emergencies, sustained investment in prevention should similarly be viewed as a strategic investment in health-system resilience, economic stability, and population wellbeing.

As the global public health community approaches the 40th anniversary of the Ottawa Charter for Health Promotion, adopted in 1986, the relevance of its core principles is increasingly evident. The Charter articulated a life-course and multisectoral vision for prevention, emphasising healthy public policy, supportive environments, community action, personal skills, and the reorientation of health services [5]. Four decades later, the persistent burden of CVD and diabetes in Europe reflects a gap not in vision, but in sustained implementation. Re-aligning contemporary NCD strategies with these foundational principles is essential to strengthening prevention across the life course.

This policy brief synthesises findings from recent WHO, EU, and international public health reports to identify key implementation gaps and policy priorities for strengthening life-course prevention of cardiovascular disease and diabetes in Europe.

## Analysis: evidence and current policy approaches

### The role of obesity, diabetes, and life-course risk accumulation

Obesity and diabetes are central drivers of cardiovascular risk in Europe, with risk trajectories often established early in life and reinforced by social, commercial, and environmental determinants. More than half of European adults and up to one third of children live with overweight or obesity, with higher prevalence among disadvantaged groups [1–3]. Diabetes is increasingly diagnosed at younger ages, extending lifetime exposure to complications and increasing long-term demand on health systems [6].

The increasing prevalence of obesity and younger-onset diabetes reflects cumulative exposure to unhealthy food environments, physical inactivity, and social disadvantage across the life course rather than isolated behavioural choices alone [3, 6]. Evidence from life-course epidemiology further demonstrates that early-life exposures substantially influence adult cardiometabolic risk trajectories and long-term cardiovascular outcomes [7].

### Evidence exists, implementation remains weak

The World Health Organization has identified cost-effective “quick buys” targeting major NCDs and shared risk factors, capable of delivering measurable population-level benefits within 5 years, including tobacco control, salt reduction, healthier food policies, and strengthened primary prevention [8]. However, the Progress Monitor 2025 indicates that implementation of these interventions remains inconsistent across Member States, particularly in relation to regulatory and fiscal measures [1]. This suggests that the persistent gap between evidence and outcomes reflects shortcomings in implementation capacity and political prioritisation rather than insufficient scientific evidence.

### Monitoring and accountability gaps

Effective prevention is further undermined by fragmented and underfunded surveillance systems. While mortality data are routinely collected, systematic monitoring of risk factors, inequalities, policy implementation, and health-system performance remains uneven across countries [1, 9]. Recent European analyses have highlighted major fragmentation in NCD surveillance systems, including inconsistent collection of equity-sensitive indicators, limited interoperability, and insufficient integration of prevention metrics into policymaking processes [9]. These limitations weaken accountability and reduce the capacity of health systems to evaluate prevention effectiveness or identify widening inequalities.

### Current policy initiatives

Recent initiatives, including the EU Safe Hearts Plan, aim to reduce premature cardiovascular mortality by 25% by 2035 and signal renewed political ambition [10]. A future evaluation of the Plan should assess its success by the extent to which it addresses underlying structural drivers of cardiovascular risk, ensures systematic monitoring of implementation and outcomes, and translates ambition into sustained, population-level prevention gains rather than continued reliance on reactive care.

## Policy options

### Embed life-course prevention across cardiovascular and diabetes strategies

Policies should explicitly integrate prevention from early childhood through older age, addressing shared risk factors for obesity, diabetes, and CVD. This includes healthier food environments, physical activity, promoting urban design, and early-life interventions that reduce long-term risk accumulation. Life-course prevention aligns with the principles of the Ottawa Charter and is likely to yield substantial health and economic benefits, but requires sustained political commitment beyond short-term cycles.

### Strengthen primary care and community-based prevention

Primary care systems should be resourced to deliver early detection, risk management, and integrated prevention for CVD and diabetes. Strong primary care supports continuity across the life course and reduces reliance on costly specialist care. Workforce capacity, training, and appropriate incentives are key facilitators, while workforce shortages and constrained financing remain major barriers.

### Invest in equity-oriented surveillance and monitoring

Surveillance systems should extend beyond mortality to include risk factors, social and commercial determinants, policy implementation, and health-system performance. Equity-sensitive, disaggregated data are essential to ensure prevention strategies reduce rather than exacerbate health inequalities. Investment in interoperable, timely data systems is critical to strengthening accountability.

### Scale collaborative implementation platforms

European Joint Actions offer practical pathways for translating policy ambition into implementation. JACARDI (Joint Action on Cardiovascular Diseases and Diabetes) supports Member States in strengthening governance, workforce capacity, integrated prevention, and care models, with an explicit focus on equity [11]. JA PreventNCD and related EU Actions emphasise integrated prevention across shared NCD risk factors. These initiatives demonstrate increasing recognition that sustainable prevention requires cross-country implementation platforms rather than isolated national strategies. Therefore, sustaining and scaling such platforms beyond time-limited project funding is essential for embedding prevention within routine public health practice [12].

### Align health, social, and economic policies

Effective prevention requires action beyond the health sector. Policies addressing social, commercial, and environmental determinants such as food systems, marketing regulation, education, and social protection are essential facilitators of long-term cardiovascular and metabolic health.

## Conclusion

Europe’s persistent burden of cardiovascular disease and diabetes reflects a failure to translate political commitment into sustained, life-course preventive action. Although evidence-based interventions and strategic frameworks are well established, their impact is constrained by weak implementation, fragmented monitoring, and continued reliance on reactive, treatment-centred care rather than prevention across the life span.

As the 40th anniversary of the Ottawa Charter approaches, its life-course and equity-oriented principles provide a timely framework for reorienting contemporary NCD strategies. Operationalising prevention from early life through older age through strong primary care, robust surveillance systems, and collaborative implementation platforms such as JACARDI and JA PreventNCD is essential to achieving meaningful and equitable reductions in premature mortality and to strengthening the long-term resilience of European health systems. In the context of ongoing discussions surrounding the future European policy and funding landscape, sustained investment in prevention should also be recognised as an investment in Europe’s economic productivity, competitiveness, and social sustainability. A competitive Europe cannot be achieved without a healthy population.
